# Enabling tobacco treatment for gastroenterology patients via a novel low-burden point-of-care model

**DOI:** 10.1186/s12913-024-11092-y

**Published:** 2024-06-20

**Authors:** Suha Abushamma, Li-Shiun Chen, Jingling Chen, Nina Smock, Giang Pham, Chien-Huan Chen

**Affiliations:** 1 Division of Gastroenterology, John T. Milliken Department of Medicine, Washington University School of Medicine, 600 S. Euclid Avenue, MSC-8124-21-427, Saint Louis, MO 63110 USA; 2 Department of Psychiatry, Washington University School of Medicine, Saint Louis, MO USA; 3 Alvin J. Siteman Cancer Center at Barnes-Jewish Hospital, Washington University School of Medicine, Saint Louis, MO USA

**Keywords:** Gastroenterology clinics, Tobacco use, Smoking cessation, Quality improvement, Electronic heath record

## Abstract

**Background & aim:**

Smoking is a major risk factor for multiple gastrointestinal cancers, and adversely affects peptic ulcer disease, gastroesophageal reflux, pancreatitis and Crohn’s disease. Despite key recommendations for diagnosing and treating tobacco use disorder in healthcare settings, the degree to which this is implemented in Gastroenterology (GI) clinics is unknown. We aimed to assess our providers’ practices, identify barriers for implementing evidence-based smoking cessation treatments, and address these barriers by implementing a novel low-burden point of care Electronic health record-enabled evidence-based tobacco treatment (ELEVATE), in GI clinics.

**Methods:**

An online survey was distributed to clinic gastroenterologists. ELEVATE module training was implemented in 1/2021. Data were evaluated during pre (7/2020-12/2020) and post (1/2021-12/2021) implementation periods to evaluate the reach and effectiveness of ELEVATE. Generalized estimating equations (GEE) were used to generate rate ratios (RR) to evaluate the intervention.

**Results:**

91% (20/22) of GI physicians responded to our survey, and only 20% often assisted patients who smoke with counseling. Lack of a systematic program to offer help to patients was reported by 80% of providers as an extremely/very important barrier limiting their smoking cessation practices. The proportion of current patients who smoke receiving cessation treatment increased from pre-ELEVATE to post-ELEVATE (14.36–27.47%, RR = 1.90, 95% CI 1.60–2.26, *p* < .001). Post-ELEVATE, 14.4% (38/264) of patients with treatment quit smoking, compared to 7.9% (55/697) of patients without treatment (RR = 1.89, 95% CI 1.26–2.82, *p* = .0021).

**Conclusion:**

Smoking practices are frequently assessed in GI clinics but barriers limiting cessation treatment exist. The use of a low burden point of care EHR enabled smoking cessation treatment module has led to a significant improvement in the treatment of smoking and subsequent cessation in our clinics. This study sheds light on an often under-recognized source of morbidity in GI patients and identifies an efficient, effective, and scalable strategy to combat tobacco use and improve clinical outcomes in our patients.

**Supplementary Information:**

The online version contains supplementary material available at 10.1186/s12913-024-11092-y.

## Introduction

Cigarette smoking is the most preventable health hazard in the United States [1], responsible for over 480,000 deaths annually [2]. About 19% of U.S. adults reported current use of a commercial tobacco product in 2020, of which cigarette smoking was the most prevalent [3]. Not only is smoking associated with an increased risk of cancers, cardiovascular and respiratory diseases, it has unfavorable effects in various gastroenterological illnesses.

Smoking is known to be an independent risk factor for the development of Crohn’s disease [4, 5], leading to a more severe disease phenotype with increased immunosuppressant use, hospitalizations, intestinal resection and postoperative infection in these patients [6–8]. Moreover, smoking cessation does not worsen clinical outcomes in ulcerative colitis [9]. Gastroesophageal reflux disease is the most common outpatient diagnosis in gastroenterology (GI) clinics [10], and patients who smoke are 2.5 times more likely to develop this condition than nonsmokers [11]. Peptic ulcer disease (PUD) is the leading etiology for GI bleeding [12], the most common inpatient GI diagnosis [13], and the prevalence of PUD in patients who smoke is doubled that of non-smokers [14]. Acute pancreatitis is another leading cause of hospitalizations in the U.S., of which patients who smoke are 1.5 times more likely to develop [15].

In addition to its role in the development of inflammatory conditions of the GI tract, smoking is implicated in the development of several GI cancers. Smoking is associated with a 1.2 times higher risk of colon cancer [16], 1.6 times higher risk of gastric cancer [17], 2.2 times higher risk of pancreatic cancer [18], and up to 2.6 times higher risk of esophageal cancer [17, 19]. Therefore, smoking cessation has been shown to be of utmost importance in the reduction of the risk of these diseases [20–23].

Smoking cessation is strongly encouraged by the American College of Gastroenterology (ACG) guideline for Crohn’s disease [24], and is a key quality measure of the Merit-based Incentive Payment System (MIPS) for Centers for Medicare & Medicaid Services (CMS) [25]. Approximately 70% of patients who smoke are interested in quitting smoking [26], but without evidence-based counseling or medication treatment the successful quit rate is less than 10% [27]. Although providers often assume patients lacked interest in treatment, studies have shown that there are more patients interested in counseling and or medications than those receiving them [28, 29].

The US Public Health Service Clinical Practice Guideline Panel recommends the 5 A’s (Ask, Advise, Assess, Assist, Arrange) as an evidence-based intervention for smoking cessation [27, 30]. The efficacy of the 5 A’s has been well established [31–34] and is further aided by the advent of the electronic health record (EHR) [35]. Evidence-based treatments for smoking cessation include behavioral support in the form of advice and counseling, nicotine replacement products, and medications [27].

Despite key recommendations and the emergence of evidence-based treatments for the management of smoking in healthcare settings, the degree to which this is implemented in GI clinics outside of inflammatory bowel disease (IBD) patients [36, 37] is unknown. Whereas smoking cessation counselors may be occasionally available in a tertiary medical center, our anecdotal experience suggests that patients usually do not want to make another trip or appointment for smoking cessation counseling. In addition, smoking cessation counseling is not reimbursed, and the availability of a smoking cessation counselor completely depends on the funding of the position. An easy and effective alternative to a smoking cessation counselor is needed for specialists in GI clinics to help patients quit smoking.

Electronic Health Record-Enabled Evidence-Based Tobacco Treatment (ELEVATE) is an innovative care model developed at our medical center to help patients quit smoking at the point of care when the traditional model of referring patients to smoking cessation counselors is not easily attainable [35, 38, 39]. This point of care model, ELEVATE, has been shown to be more cost-effective than the traditional referral to a smoking cessation specialist model in our oncology clinics where the tobacco-cancer link is more salient [40]. However, GI patients without a cancer diagnosis may not have the same urgency and motivation to quit smoking. In addition, except for IBD patients, GI providers are guided by GI societies to assess and treat smoking, unlike the direct recommendations stated in the National Comprehensive Cancer Network (NCCN) Clinical Practice Guidelines in Oncology (nccn.org). Therefore, it is not clear whether the success of this low-burden care model in oncology clinics can be extrapolated to patients and providers in GI clinics.

In this study, we aimed to (a) assess our providers’ practices and identify perceived barriers for implementing evidence-based smoking cessation treatments, and (b) implement an evidence-based EHR-assisted point-of-care smoking cessation module in our GI clinics.

## Methods

### Design and setting

This is a quality improvement initiative aimed at smoking cessation performed at a major tertiary medical center’s outpatient GI clinics. A survey was distributed to our GI attending physicians with outpatient clinics regarding their smoking cessation care practices and potential barriers We then implemented a point of care smoking cessation module intervention in our GI clinics, known as the Electronic Health Record-Enabled Evidence-Based Tobacco Treatment (ELEVATE) program, and evaluated its reach and effectiveness with pre- and post- comparisons. Our electronic health record (EHR) system is EPIC (Madison, Wisconsin). The data for reach and effectiveness was extracted from the EHR.

### Provider survey

Our GI attending physicians with outpatient clinics were invited to anonymously complete an 11-question online survey in 2020 that assessed: (1) smoking treatment practices e.g. (In the past month, how often did you advise patients who smoked to quit smoking) on a 5-point Likert scale (Never, rarely, sometimes, often, always), and (2) potential barriers to implementing smoking treatment practices. Multiple barriers were listed and providers were asked to rate each item separately on a 5-point Likert scale (not at all important, not so important, somewhat important, very important, extremely important). The survey assessed the components of the 5 A’s (Ask, Advise, Assess, Assist, Arrange). It was adapted from the smoking knowledge, attitudes, and practices (S-KAP) instrument [41], and has been validated in a previous study [29] (Supplementary Text S1).

### Implementation of ELEVATE

#### Workflow

The ELEVATE intervention has been implemented successfully at our Oncology clinics and described in a prior publication by Ramsey et al. [35]. ELEVATE leverages EPIC EHR functionality to ensure consistent tobacco use assessment and cessation treatment support is provided for all our clinic patients utilizing the 5 A’s, as illustrated in Supplementary Fig. 1. ELEVATE has been featured by EpicShare as a Share & Learn for implementing tobacco treatment [42]. ELEVATE supports a team-based approach to activate the medical team including medical assistants and physicians. This point of care workflow begins when the medical assistant *Asks* about tobacco use and *Advises* cessation using a brief script: “The best thing you can do for your health is quit smoking.” For all patients who smoke and have not been offered counseling options in the preceding 90 days, a Tobacco Intervention Best Practice Advisory (BPA) embedded in the EHR prompts the medical assistant to *Assess* patient motivation and *Assist* the patient with referrals to additional tobacco treatment via phone-based (Quitline), SMS text-based (SmokefreeTXT), or smartphone app-based (QuitStart) counseling. If phone-based or SMS test-based counseling is chosen, the referral will be sent electronically by the EHR based on the patient’s choice of program. If smartphone app-based counseling is chosen, directions for application download and set-up are printed and provided to the patient. Furthermore, another BPA prompts the physicians to prescribe evidence-based tobacco cessation medications (nicotine replacement therapy, bupropion, or varenicline). Finally, nicotine dependence is added as part of the BPA in the problem list for ongoing tracking and follow up point of care treatment.

#### Training

The GI clinic rooming staff (medical assistants) received training on how to utilize ELEVATE in the beginning of January, 2021. Training consisted of both online and in-person seminars by our team members, Gastroenterology Fellow (S.A.) and Program Manager (N.S.). Based on the team-based approach where medical assistants assess smoking status, provide brief advice based on a standardized script, offer referral to additional smoking cessation counseling, and pend the referral order, detailed training was provided only to medical assistants. The GI clinic physicians were informed about this Quality Initiative and advised to approve the referral orders pended by their medical assistants.

#### Data extraction and outcomes

As part of Quality Improvement, data was extracted from EPIC for 6 months prior to ELEVATE training (7/2020–12/2020), 6 months post-training (1/2021–6/2021) and the ensuing 6 months (7/2021 -12/2021) to assess sustainability over time. Outcomes were defined using the entire EHR data and same criteria as previously published [35, 38, 39]. Reach is defined as % of patients receiving evidence based treatment (brief advice, referral to counseling, or pharmacotherapy such as nicotine replacement, bupropion or varenicline) among patients who smoke. Effectiveness is defined as % of patients who quit smoking in the subsequent 6 months among patients who smoke (e.g., patients who smoke changed from current smoker to former smoker during subsequent healthcare visits).

### IRB approval

We submitted our project to the hospital’s institutional review board (IRB). As this study was part of a quality improvement project and was not designed to answer a research question or test a hypothesis, it was not considered to meet federal definitions and was given an exempt determination by the IRB.

### Statistical analysis

Data analysis entailed comparisons between pre/post-ELEVATE implementation proportions of patients receiving a tobacco use assessment, patients identified as smokers who were given advice about cessation, and patients identified as smokers who were prescribed or documented as using cessation medications.

Generalized estimating equations (GEE) were used to generate rate ratios (RR) to evaluate the intervention. These GEE modules adjusted for repeated patients appearing in different time blocks, as well as demographic covariates of age, sex, and race.

All statistics were performed using SAS, version 9.3 for Microsoft Windows (SAS Institute, Inc. Cary, NC).

## Results

### Providers’ perspectives: lack of a systematic program to offer help

**Fig. 1:**
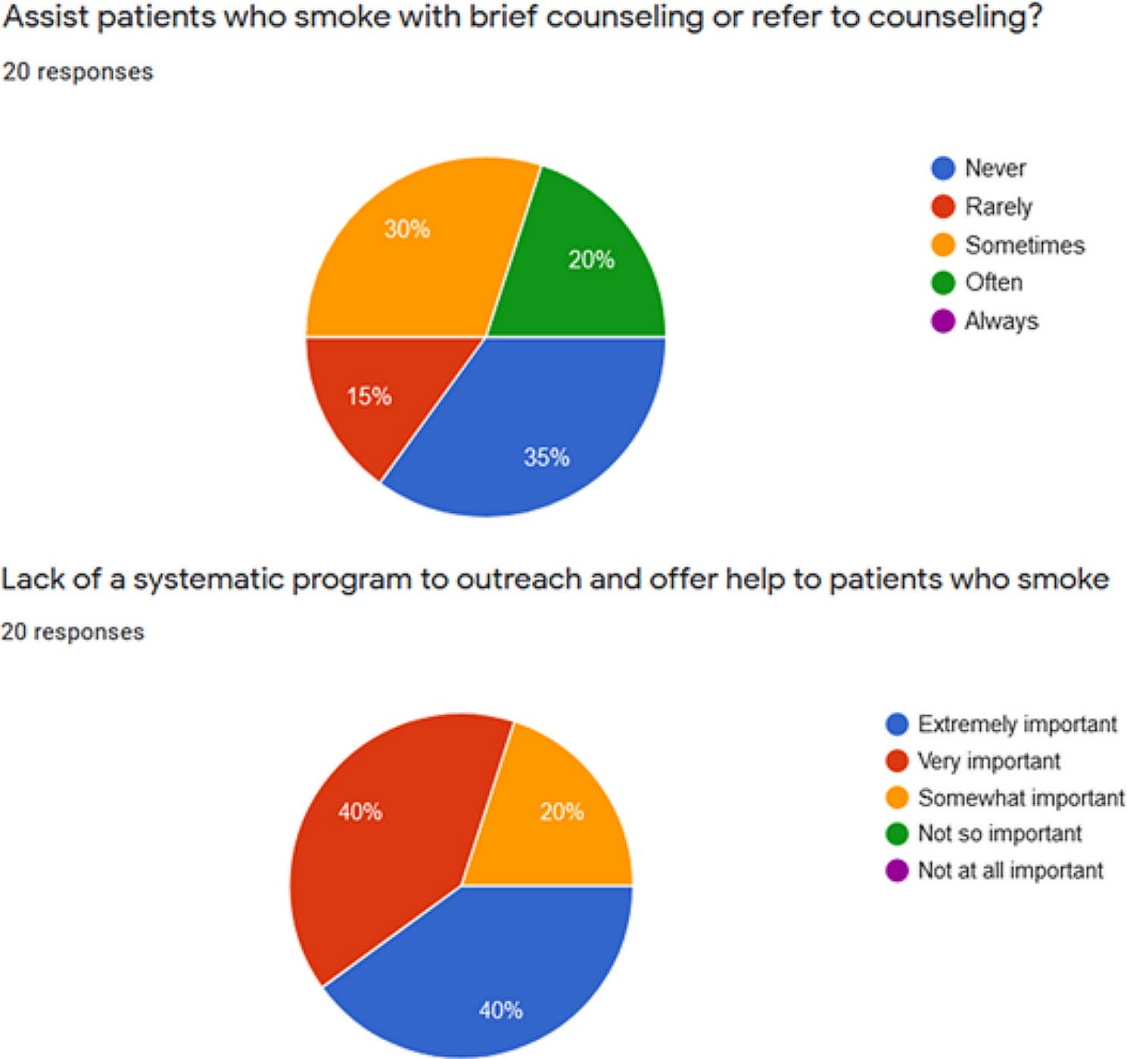
Top: Survey Question: In the past month, how frequently did you assist patients who smoke with brief counseling or refer to counseling? Bottom: Survey Question: Please rate the importance of the following that might limit your smoking cessation treatment practice: Lack of a systematic program to outreach and offer help to patients who smoke

Most (91% 20/22) attending GI physicians responded to our anonymous survey regarding smoking cessation practices in their outpatient clinics, and nearly half (45%) of them stated they always asked their patients if they smoked. Although 75% of the physicians stated they would assess their patients’ willingness to quit at the time of the visit more often than not, only 20% often assisted patients who smoke with counseling (Figs. 1), 10% had ever assisted patients by prescribing smoking cessation medications, and 85% rarely or never arranged a follow-up plan to discuss smoking and quitting. When asked about barriers limiting their smoking cessation treatment practices, 85% of the providers reported “Patients not interested” as an extremely/very important factor, followed by the “Lack of a systematic program to outreach and offer help to patients who smoke”, reported by 80% of providers as an extremely/very important factor (Fig. 1).

### Decision support tools (ELEVATE module) led to increased tobacco use assessment and treatment

#### Tobacco use assessment

Out of the 8259 patients who had visited our GI outpatient clinics in the 6 months pre-ELEVATE module training, 88.9% (7349) were assessed for smoking. This is in comparison to 91.5% (7861/8587) of the patients who had visited our clinics in the 6 months post-ELEVATE training (RR = 1.02, 95% CI 1.02–1.03, *p* < .0001).

#### Tobacco treatment

Treatment consisting of behavioral support in the form of brief advice given, counseling offered or counseling referred had significantly increased from 0.6 to 12%, 0.6–34% and 0.6–8% respectively, post-ELEVATE training (Table 1). Provision of any treatment increased 91% post-ELEVATE training. Here, we note no change in cessation medication prescription as the current initiative phase targeted the rooming staff who did not prescribe medication.

**Table 1:** Smoking prevalence and treatment among GI patients pre-ELEVATE module training vs. post-ELEVATE

		Pre-ELEVATE training (7/2020 -12/2020) (*n* = 8259)			Post-ELEVATE training (1/2021-6/2021) (*n* = 8587)						
		*N*	*n*	%	*N*	*n*	%	RR	Relative % Increase	95%CI	*p*
Assessment		8259	7349	88.98	8587	7861	91.55	1.02	3	1.02–1.03	< 0.0001
Any Treatment*		989	142	14.36	961	264	27.47	1.90	91	1.60–2.26	< 0.0001
Brief Advice		989	6	0.61	961	118	12.28	20.36	1913	8.97–46.24	< 0.0001
Additional Counseling Offered		989	6	0.61	961	326	33.92	55.87	5460	25.07-124.53	< 0.0001
Additional Counseling Referred		989	6	0.61	961	77	8.01	13.70	1213	6.08–30.89	< 0.0001
Medication		989	136	13.75	961	112	11.65	0.84	N/A	0.69–1.03	0.0993

*Any treatment is defined as patients receiving brief advice, medication, additional counseling offered, or additional counseling referred

### Decision support tools (ELEVATE module) has sustained effect

**Fig. 2:**
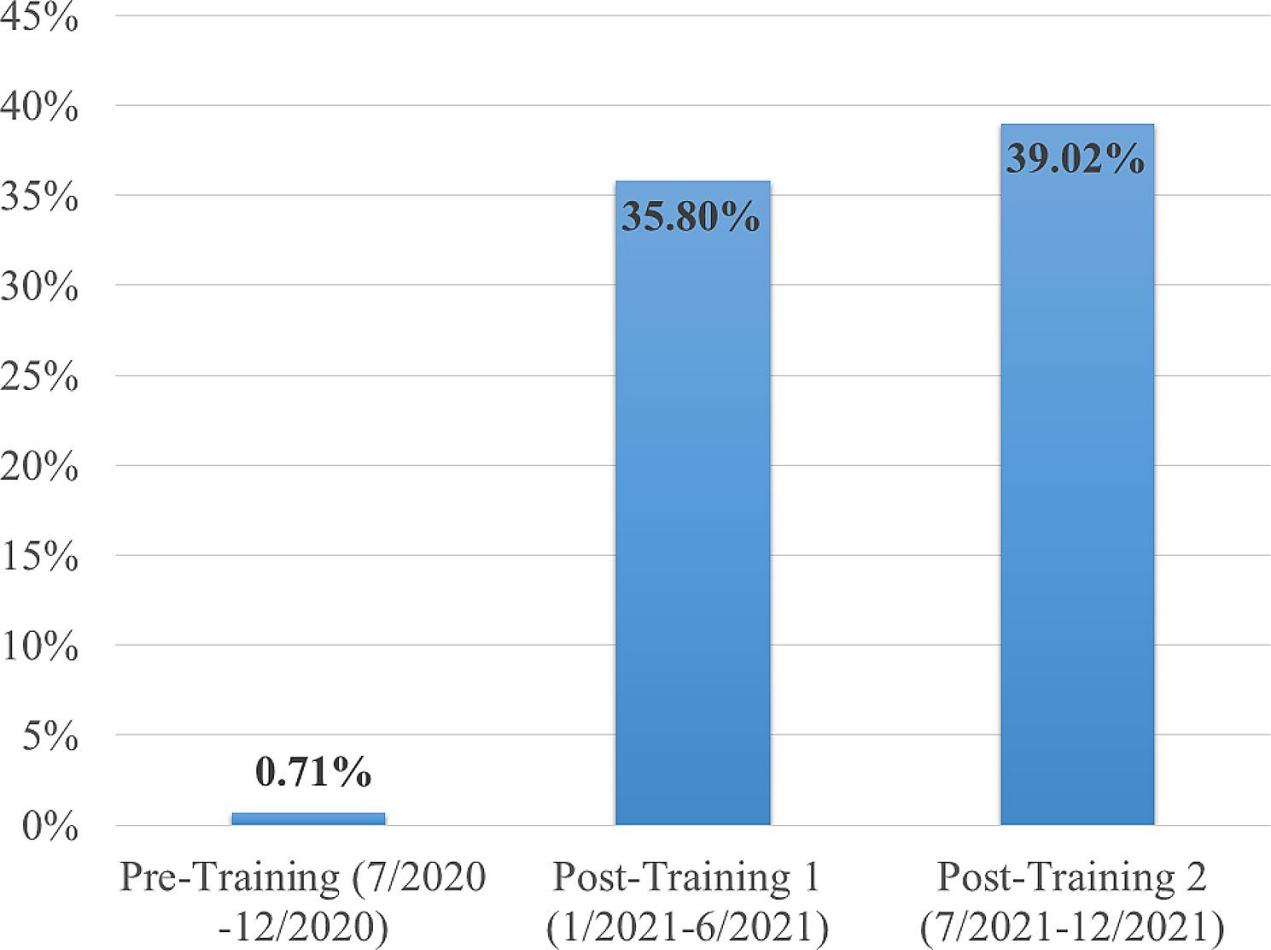
Pre/Post-ELEVATE module training tobacco treatment (behavioral support) and sustainability over time

The effect of ELEVATE on tobacco assessment and treatment (behavioral support) has sustained in these clinics not only in the 6 months after implementation (post-training 1), but also 12 months after the implementation (post-training 2) (Supplementary Fig. 2 and Fig. 2). Specifically, tobacco assessment remains at 96% in post-training 2, compared to 92% in post-training 1 and 89% in the pre-training period. Similarly, the provision of behavioral support (brief advice, offer and referral of additional counseling options) were 0.7%, 36%, and 39% during the pre-training period, post-training 1, and post-training 2 respectively. Tobacco assessment and behavioral support were both significantly higher in post-training 2 compared with the pre-training period (96% vs. 89%, RR = 1.07, 95% CI 1.04–1.11, *p* < .0001 for tobacco assessment; 39% vs. 0.7%, RR = 54.93, 95% CI 26.16-115.37, *p* < .0001 for behavioral support).

### Gastroenterology patients given tobacco treatment had more success in smoking cessation

**Fig. 3:**
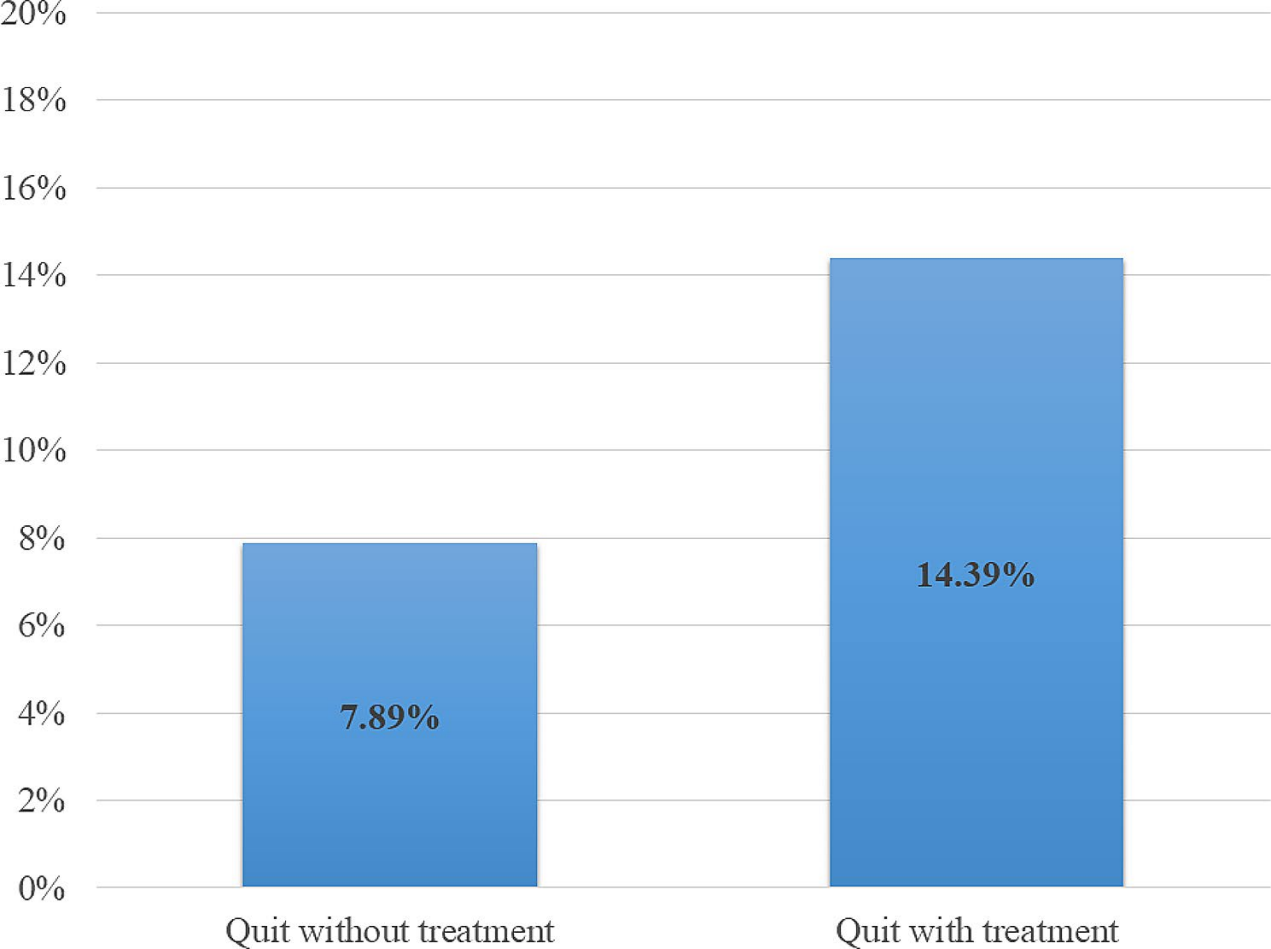
Post-ELEVATE module training tobacco treatment effectiveness

Patients who received treatment had higher success in quitting smoking compared to those who did not receive treatment. For example, 14.4% (38/264) of patients with treatment quit smoking, compared to 7.9% (55/697) of patients without treatment in post-training period 1 (RR = 1.89, 95% CI 1.26–2.82, *p* = .0021) (Fig. 3). We observed an increase in the overall quit rates amongst all patients (treated and untreated) in pre vs. post periods, although it did not reach statistical significance (8.2% vs. 9.7%, RR = 1.22, 95% CI 0.95–1.57, *p* = .12), as the majority of patients did not receive any treatment.

## Discussion

To our knowledge, this is the first study to assess smoking cessation practices and apply a successful smoking cessation treatment program across all outpatient GI clinics including sub-specialties such as IBD, hepatology, and interventional endoscopy. Evidence-based tobacco treatment has been rarely delivered to gastroenterology patients despite its clinical benefit based on our survey. Our study identified the lack of a systematic program to help patients who smoke as an important barrier preventing the delivery of tobacco treatment in GI clinics. To address this barrier, we implemented a low-burden point-of-care model via EHR-enabled health informatics tools that resulted in a sustained increase in the reach of evidence-based tobacco treatment in a large academic gastroenterology practice.

Most gastroenterologists want to help patients who smoke but face multiple barriers. Lack of patient interest in smoking cessation was reported by 85% of our gastroenterologists as a factor limiting cessation treatment, which was perceived as an important barrier and previously described by 76% of general practitioners [43] and 91% of psychiatrists [29]. Contrary to physician’s perceptions, studies have shown that almost half of patients who smoke were interested in quitting while only a few of them were on treatment [29, 44]. This perceived lack of interest in smoking cessation may partly explain the noted difference. Although over two thirds of our gastroenterologists usually asked patients about whether they smoked, less than half of them consistently advised patients to quit smoking, and even less prescribed cessation medications. A similar finding was reported by Williams et al. [45], where 80% of providers often asked about smoking status but treatment was provided in less than 30% of patients who smoke. This is important as a Cochrane review of 17 trials noted that patients who smoke who received only brief advice to quit from their physicians without other treatment were 1.6 times more likely to quit than those who did not [46]. In our study, we have observed a substantial quit rate (14.4%) among patients who received treatment, which is much higher than the low (∼ 5%) quitting success rate among individuals who try to quit smoking on their own [47].

These findings suggest the promise and impact of efficiently incorporating tobacco treatment into gastroenterology care using a team-based approach by including team members at all levels. Smoking cessation advice does not necessarily need to come from physicians. A large Cochrane review with over 17,000 patients highlighted the effectiveness of nurses in smoking cessation counseling [48], and the important role medical assistants play in smoking cessation interventions has been well described previously [49, 50]. Therefore, the US Public Health Service Clinical Practice Guideline Panel recommends a multidisciplinary team approach to smoking cessation interventions that can include physicians, nurses, medical assistants, and other team members [27]. In our study, smoking cessation advice was delivered by medical assistants at the time of rooming, incorporated into regular workflow and supported by the EHR functions.

Clinic staff training is paramount when launching smoking cessation interventions, as noted by Sheffer et al. where training increased the use of a fax referral program more than five folds compared to no training [51], and Williams et al. who reported doubling of tobacco use assessment post training [44]. ELEVATE module training was provided to our clinic staff in 1/2021, which led to a significant increase in tobacco use assessment, and almost twofold increase in tobacco cessation treatment, a change that was sustained for up to 1 year after.

Implementing evidence-based treatment will benefit from an efficient workflow with decision support enabled by health informatics tools. As a low-burden tobacco treatment model, ELEVATE results in high reach and comparable effectiveness to other care models [38–40, 52]. The smoking cessation rate with ELEVATE, about 15%, is comparable to the smoking cessation rate with a smoking cessation counselor in the previously published studies [53–57].

Patient safety and quality improvement is very important but can be costly to implement. The ELEVATE program only requires adding an informatics tool to the EHR and staff training. It is low cost and does not require funding of a smoking cessation counselor. Since behavioral support treatment is provided by medical assistants during the standard rooming process, it adds little to the workload burden of medical assistants. In fact, anecdotally our medical assistants appreciated this opportunity of contributing to the care of patients. The ELEVATE program has been implemented successfully in our oncology clinic since 2018, but it was unknown whether GI patients without a cancer diagnosis would be motivated and receptive to the program. Our findings suggest that a systematic approach such as ELEVATE can be adapted to GI clinics, and potentially other specialties where tobacco use affects clinical outcomes.

In the current medical environment, providers are under pressure to see more patients with less time. Therefore, low burden programs such as ELEVATE should be implemented, evaluated, and improved to help to facilitate tobacco treatment for all providers within a given clinic. Our study shows how training of medical assistants using a team approach can significantly increase behavioral support treatment for smoking cessation, which includes brief advice given, counseling offered and/or referred.

These findings also revealed an important future direction. The current phase of ELEVATE targeted the rooming staff (e.g., medical assistants) and enabled their offer of behavioral support during every gastroenterology encounter. Behavioral support alone has been shown to be effective in smoking cessation, as reported by Carpenter et al. who noted no differences in cessation attempts between patients who smoke receiving behavioral support with nicotine replacement therapy compared to those receiving behavioral support only [58], and Aveyard et al. who noted an over twofold increase in the frequency of quit attempts with behavioral support only [59]. Consistent with previous studies, we found an almost twofold increase in smoking cessation with treatment after ELEVATE implementation and clinic staff straining.

Although the ELEVATE program increased the behavioral supportive treatment for smoking cessation, more than half of the patients remained untreated 1 year after implementation. Part of the reason could be that the BPA is not a hard stop in the workflow. This underscores the importance of continued staff training to further increase the incorporation of treatment in the workflow of medical assistants.

Since the ELEVATE program of our GI clinics focused at this stage on training medical assistants to provide behavioral support treatments, we observed no change in the rate of medications prescription, another important treatment component for patients who smoke. The low medication prescription rate is in line with multiple previously reported studies [60–62], and reflects that most gastroenterologists may not have the time or be as comfortable prescribing these medications, a potential leverage point for future tobacco interventions. At the next stage, we plan to further expand the EHR algorithm to incorporate prescribing medical therapies for smoking cessation.

Another important aspect of ELEVATE is its cost-effectiveness potential. The cost of ELEVATE is ∼$70 per patient and reducing when it is scaled up to more clinics with minimal cost increase because it relies on regular staff to provide point of care tobacco treatment. ELEVATE is the most cost-effective program among many tobacco programs in the cost effectiveness analyses for tobacco programs in the Cancer Moonshot Initiative [40, 63]. In gastroenterology, the cost saved by smoking cessation in a patient with Crohn’s disease for example, is estimated to be in the range >$1,000 per patient given tobacco-related treatment failure, hospitalizations, and surgeries [64].

The results of our study should be interpreted in the context of several limitations. First, as with any study with surveys, there is a potential for recall bias. However, we limited our questions to only one month prior to the survey, and provided answer options in an ordinal scale, rather than requiring exact quantitative answers. Second, our intervention was implemented in a single institution’s GI clinics, and thus the results may differ when applied in other clinics with different patient populations. However we do not believe this would alter our results significantly, as the ELEVATE module has been implemented in our institution’s oncology clinics, with similar increases in tobacco treatment reach and effectiveness [38]. Third, this was not a randomized trial with control clinics without the intervention, therefore we cannot rule out the influence of temporal trends such as COVID-19’s effect on increased smoking cessation, although current literature suggests an increase in tobacco use during Covid-19 related to overall increase of isolation and stress [65]. Fourth, the outcome of smoking cessation effectiveness was captured by the EHR smoking status of which accuracy can be limited due to patient self-report, rooming staff documentation, and update frequency in busy healthcare settings. We have tried to minimize this bias in training the rooming staff for the workflow to “assess” smoking status in every encounter instead of “keeping” the documented smoking status from prior encounters. To reduce this bias, we have emphasized this workflow in our recurrent training for the rooming staff and ongoing monitoring of the EHR data that shows actual transition of smoking status from encounter to encounter. Fifth, we have coded treatment as positive if patients received treatment in any of the encounters during that time frame and it’s possible for patients to receive tobacco treatment repeatedly in more than one encounter. Future research should evaluate the frequency of treatment and its potential dose response effect on patient outcomes. Sixth, we have not controlled for patients’ socioeconomic status which potentially influence patients’ receipt of tobacco treatment. Finally, smoking cessation was not verified biochemically, albeit validation is not typically required in low intensity non-trial and large EHR-based interventions such as this study [66]. 

In summary, this study examined smoking cessation practices in a large tertiary center’s GI clinics, identified potential barriers to treatment, and implemented an EHR enabled evidence-based smoking cessation treatment program. ELEVATE program is effective, low cost, sustainable, and can be applied to GI clinics, ultimately improving clinical outcomes in our patients.

### Electronic supplementary material

Below is the link to the electronic supplementary material.

Supplementary Material 1

Supplementary Material 2

Supplementary Material 3

## Data Availability

The datasets generated and/or analysed during the current study are not publicly available as they were generated from our institution’s electronic medical record, but are available from the corresponding author on reasonable request.
