# Supplementary material for: Enabling tobacco treatment for gastroenterology patients via a novel low-burden point-of-care model
Source: BMC Health Serv Res. 2024 Jun 20;24:752. doi: 10.1186/s12913-024-11092-y (PMC11188289; doi:10.1186/s12913-024-11092-y)
Supplement: Supplementary file 1 — Supplementary Material 1 [file 12913_2024_11092_MOESM1_ESM.docx]

[Practice] In the past month, how frequently did you:

* Required

1. Ask your patients whether they smoked? *

*Mark only one oval.*

Never Rarely Sometimes Often Always

1. Advise patients who did smoke to quit smoking? *

*Mark only one oval.*

Never Rarely Sometimes Often Always

1. Assess if patients are willing to attempt to quit at this time? *

*Mark only one oval.*

Never Rarely Sometimes Often Always

1. Assist patients who smoke with medication? *

*Mark only one oval.*

Never Rarely Sometimes Often Always

1. Assist patients who smoke with brief counseling or refer to counseling? *

*Mark only one oval.*

Never Rarely Sometimes Often Always

1. Arrange a follow-up plan to discuss smoking and quitting? *

*Mark only one oval.*

Never Rarely Sometimes Often Always

[Barrier] Please rate the importance of the following that might limit your smoking cessation treatment practice:

1. Patients not interested *

*Mark only one oval.*

Extremely important Very important Somewhat important Not so important Not at all important

1. Lack of provider time *

*Mark only one oval.*

Extremely important Very important Somewhat important Not so important Not at all important

1. Lack of community resources to refer patients *

*Mark only one oval.*

Extremely important Very important Somewhat important Not so important Not at all important

1. Lack of training *

*Mark only one oval.*

Extremely important Very important Somewhat important Not so important Not at all important

1. Lack of a systematic program to outreach and offer help to patients who smoke *

*Mark only one oval.*

Extremely important Very important Somewhat important Not so important Not at all important

*Skip to question 12*

Additional Comments

1. Thank you for helping improve our care! Feel free to share any additional comments here
