# Supplementary figures and images for: Enabling tobacco treatment for gastroenterology patients via a novel low-burden point-of-care model
Source: BMC Health Serv Res. 2024 Jun 20;24:752. doi: 10.1186/s12913-024-11092-y (PMC11188289; doi:10.1186/s12913-024-11092-y)

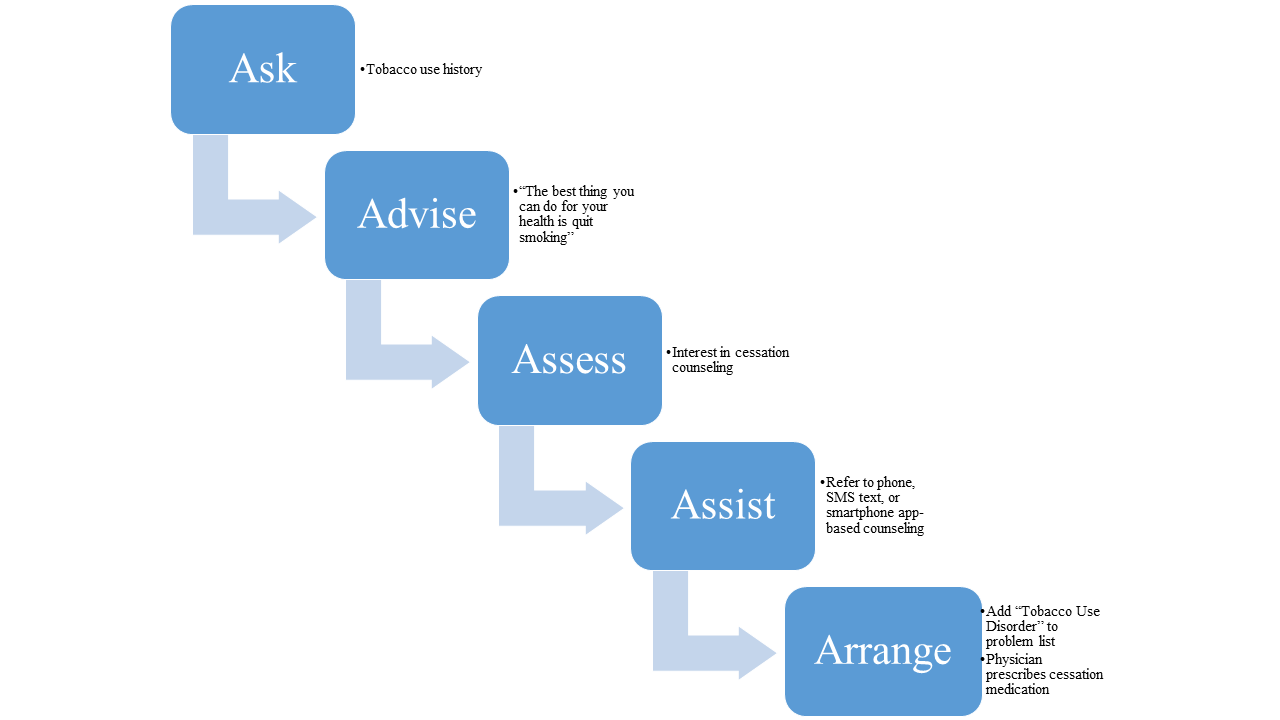

Supplement: Supplementary file 2 — Supplementary Material 2 [file 12913_2024_11092_MOESM2_ESM.png]

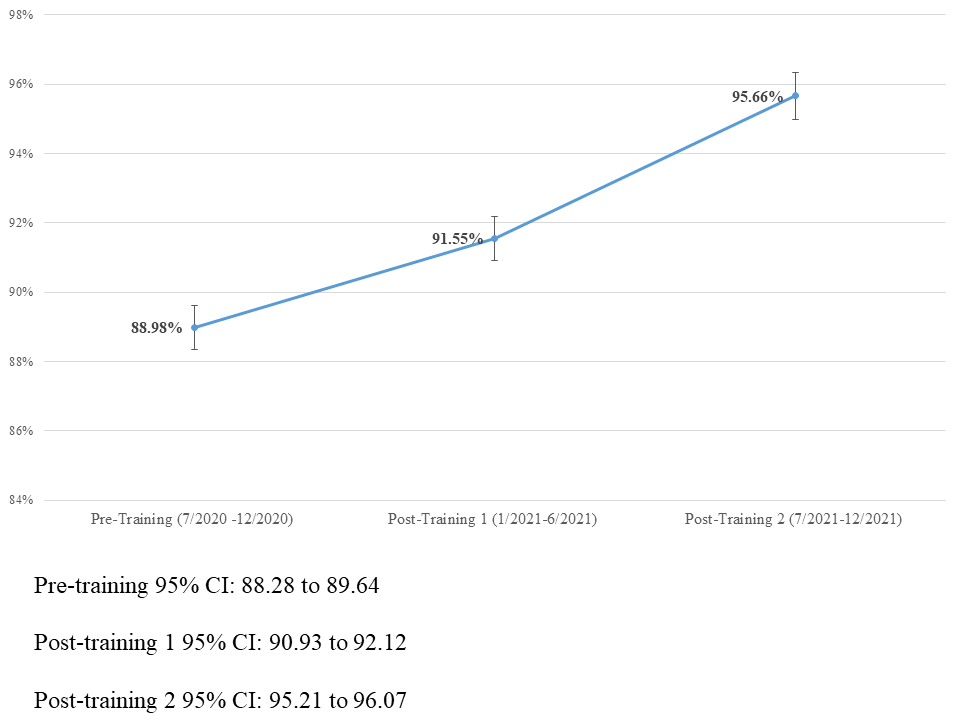

Supplement: Supplementary file 3 — Supplementary Material 3 [file 12913_2024_11092_MOESM3_ESM.png]
